# Serum secretoneurin as a promising biomarker for predicting poor prognosis in intracerebral hemorrhage: A prospective cohort study

**DOI:** 10.1007/s10143-024-02566-y

**Published:** 2024-07-13

**Authors:** Xutong Zhu, Hao Shan, Zefan Wang, Yucheng Wang, Tian Yan, Ziyin Chen, Xin Zhang

**Affiliations:** 1 The First School of Clinical Medicine, Zhejiang Chinese Medical University, No. 548 Binwen Road, Hangzhou, 310053 China; 2 The Fourth School of Clinical Medicine, Zhejiang Chinese Medical University, No. 548 Binwen Road, Hangzhou, 310053 China; 3 Department of Neurosurgery, The First Affiliated Hospital of Zhejiang Chinese Medical University, No. 54 Youdian Road, Hangzhou, 310006 China

**Keywords:** Secretoneurin, Intracerebral hemorrhage, Biomarkers, Disease severity, Prognosis

## Abstract

**Objective:**

Secretoneurin may play a brain-protective role. We aim to discover the relationship between serum secretoneurin levels and severity plus neurological outcome after intracerebral hemorrhage (ICH).

**Methods:**

In this prospective cohort study, serum secretoneurin levels were measured in 110 ICH patients and 110 healthy controls. Glasgow Coma Scale (GCS) and hematoma volume were used to assess stroke severity. Poor prognosis was defined as Glasgow Outcome Scale (GOS) scores of 1–3 at 90 days after ICH. A multivariate logistic regression model was constructed to determine independent correlation of serum secretoneurin levels with severity and poor prognosis. Under receiver operating characteristic (ROC) curve, prognostic ability of serum secretoneurin levels was assessed. Restricted cubic spline (RCS) model and subgroups analysis were used for discovering association of serum secretoneurin levels with risk of poor prognosis. Calibration curve and decision curve were evaluated to confirm performance of nomogram.

**Results:**

Serum secretoneurin levels of patients were significantly higher than those of healthy controls. Serum secretoneurin levels of patients were independently correlated with GCS scores and hematoma volume. There were 42 patients with poor prognosis at 90 days following ICH. Serum secretoneurin levels were significantly higher in patients with poor outcome than in those with good outcome. Under the ROC curve, serum secretoneurin levels significantly differentiated poor outcome. Serum secretoneurin levels ≥ 22.8 ng/mL distinguished patients at risk of poor prognosis at 90 days with a sensitivity of 66.2% and a specificity of 81.0%. Besides, serum secretoneurin levels independently predicted a 90-day poor prognosis. Subgroup analysis showed that serum secretoneurin levels had non-significant interactions with other variables. The nomogram, including independent prognostic predictors, showed reliable prognosis capability using calibration curve and decision curve. Area under the curve of the predictive model was significantly higher than those of GCS scores and hematoma volume.

**Conclusion:**

Serum secretoneurin levels are strongly related to ICH severity and poor prognosis at 90 days after ICH. Thus, serum secretoneurin may be a promising prognostic biomarker in ICH.

## Introduction

Intracerebral hemorrhage (ICH) is defined as the entry of hemorrhage into brain parenchyma and is characterized by high morbidity and high mortality [1]. After ICH, besides direct brain damage by hematoma, blood-brain barrier disruption, microvascular failure and cerebral edema can be induced via a series of cascading reactions, including oxidative stress, inflammatory reaction and apoptosis, thereby leading to neurological dysfunction and even death of such patients [2]. Glasgow coma scale (GCS) and hematoma volume are believably applied as the two prognostic indicators of ICH [3]. Interestingly, some biomarkers, such as S100B protein, neuron-specific enolase and glial fibrillary acidic protein, have been extensively studied as predictors of ICH to evaluate disease severity and predict prognosis [4, 5].

Secretoneurin, a 33-amino acids neuropeptide, is identified as a member of the granin family. It is mainly located in the brain [6]. At the state of tissue hypoxia, secretoneurin expressions were greatly enhanced. Moreover, angiogenesis is recognized as a key protective mechanism [7]. Experimental data on ischemic stroke, neonatal hypoxic-ischemic encephalopathy, epilepsy and Alzheimer’s disease indicated that secretoneurin may functionally act as a brain-protective factor [8–11]. Interestingly, serum secretoneurin levels were significantly elevated after ischemic stroke and traumatic brain injury [12, 13]. Noteworthily, increased serum secretoneurin levels were highly correlated with disease severity and poor prognosis of such patients. Hence, secretoneurin might be a potential biomarker of acute brain injury. Here, a prospective cohort study was conducted to investigate the prognostic significance of serum secretoneurin in ICH.

## Materials and methods

### Study population

In this prospective cohort study, we consecutively recruited ICH patients who were admitted to the Affiliated Hangzhou First People’s Hospital, Westlake University School of Medicine (Hangzhou, China) between August 2021 and August 2023. We required that all patients should be hospitalized within 24 h of the ICH onset, be at least 18 years old and underwent non-surgical treatments for hematoma. Afterwards, we excluded (1) patients with previous or current neurological disorders, such as brain tumor, encephalitis, ischemic stroke, traumatic brain injury and neurodegenerative disease; (2) those with ICH resulting from arteriovenous malformations, moyamoya’s disease, head trauma and hemorrhagic transformation of ischemic stroke; (3) those with severe diseases in other organs, such as infections, heart failure, cirrhosis and hyperthyroidism; (4) those with some specific conditions, such as pregnancies, insufficient information, rejection to participation, loss to follow up and unqualified samples. The control group consisted of healthy subjects, who were recruited simultaneously. This study was conducted in accordance with the ethical guidelines of the Declaration of Helsinki, and its protocol was approved by the Institutional Review Board of the Affiliated Hangzhou First People’s Hospital, Westlake University School of Medicine (Approval number, Medical Ethics Review No. (058)-01). Written informed consent was obtained from patients’ next of kin and controls themselves.

### Data collection

The collected information, including general demographic information (age and gender), peripheral blood pressures (systolic arterial blood pressure and diastolic arterial blood pressure), current smoking, alcohol consumption and chronic diseases (hypertension and diabetes mellitus), were recorded. Moreover, the GCS at admission was used to evaluate the severity of ICH. Based on computed tomography (CT) imaging, hematoma locations were divided into supratentorial and subtentorial cavities. Intraventricular extension of hematoma was observed. Hematoma volume was calculated using the Coniglobus formula (V = A×B×C×1/2) [14]. The Glasgow Outcome Scale (GOS) at 90 days after ICH was applied as a criterion for evaluating the 90-day clinical outcome of ICH. The scores of 1–3 signified a poor prognosis [15].

### Immune experiment

The peripheral blood specimens, collected from subjects at admission and controls at entry into the study, were used for laboratory tests. Blood glucose, blood sodium, blood potassium, blood leucocyte count and blood C-reactive protein were measured using the conventional methods. Samples were centrifuged at 3000×g for 10 min, and the serum was placed in a refrigerator at -80 °C for storage. Serum secretoneurin levels were measured by Enzyme-linked Immunosorbent Assay (ELISA). This ELISA kit was purchased from Phoenix Biotech Co., Ltd (catalog number, EK-047-95; Beijing, China). The detection range was from 0.11 to 9.8 ng/mL. Both the intra-assay coefficient of variation and the inter-assay coefficient of variation were less than 10%. Following the manufacturer’s instructions, serum secretoneurin levels were in duplicate gauged by the same technician, who was inaccessible to clinical information. The double measurements were averaged for final analysis.

### Statistical analysis

Statistical Package for Social Sciences Version 25.0 (IBM Corporation., Armonk, NY, USA) was used for statistical analysis. Figures were plotted using the packages of ggplot, forestplot, rms, nomogramFormula, pROC and rmda (R environment, version 4.2.2). Qualitative data were reported as counts (percentages). Normal distribution of quantitative data was tested by Kolmogorov-Smirnov test or Shapiro-Wilk test as appropriate. Normally distributed data were summarized as mean ± standard deviation. Median (upper-lower quartiles) were shown for skewed distributed data. Comparisons of two groups in qualitative data were performed by the χ2 test or Fisher’s exact test as appropriate. Unpaired t-test and Mann-Whitney U test were used to assess intergroup variability in normally distributed data and skewed distributed data respectively. Using the Kruskal-Wallis H test, serum secretoneurin levels were compared among multiple groups, which were divided using GOS scores. For assessing bivariate correlations, the Spearman correlation coefficients were used. Subsequently, the multivariate linear regression model was built to clarify independently correlated factors with serum secretoneurin levels. To find independent factors, which were associated with poor outcomes, we established a binary logistic regression model, in which odds ratios (ORs) and 95% confidence intervals (CIs) were reported. Under receiver operating characteristic (ROC) curve, area under curve (AUC) was calculated to assess the prognostic predictive accuracy of serum secretoneurin levels. Restricted cubic spline (RCS) model with four knots was applied to explore the relationship between serum secretoneurin levels and the risk of poor prognosis. Afterwards, subgroup analysis was conducted to determine interactions with age, gender, current smoking, alcohol consumption, hypertension and diabetes mellitus. The significant differences and interactions of subgroups were graphically represented by forest plot. Subsequently, nomogram analysis, which contained scoring system and prediction system, was developed to predict the risk of adverse prognosis. Calibration curve and decision curve were configured to show the stability and clinical benefit of the prognosis prediction. Statistically significant differences were set at *P* < 0.05.

## Results

### Selection and characteristics of patients

**Fig. 1:**
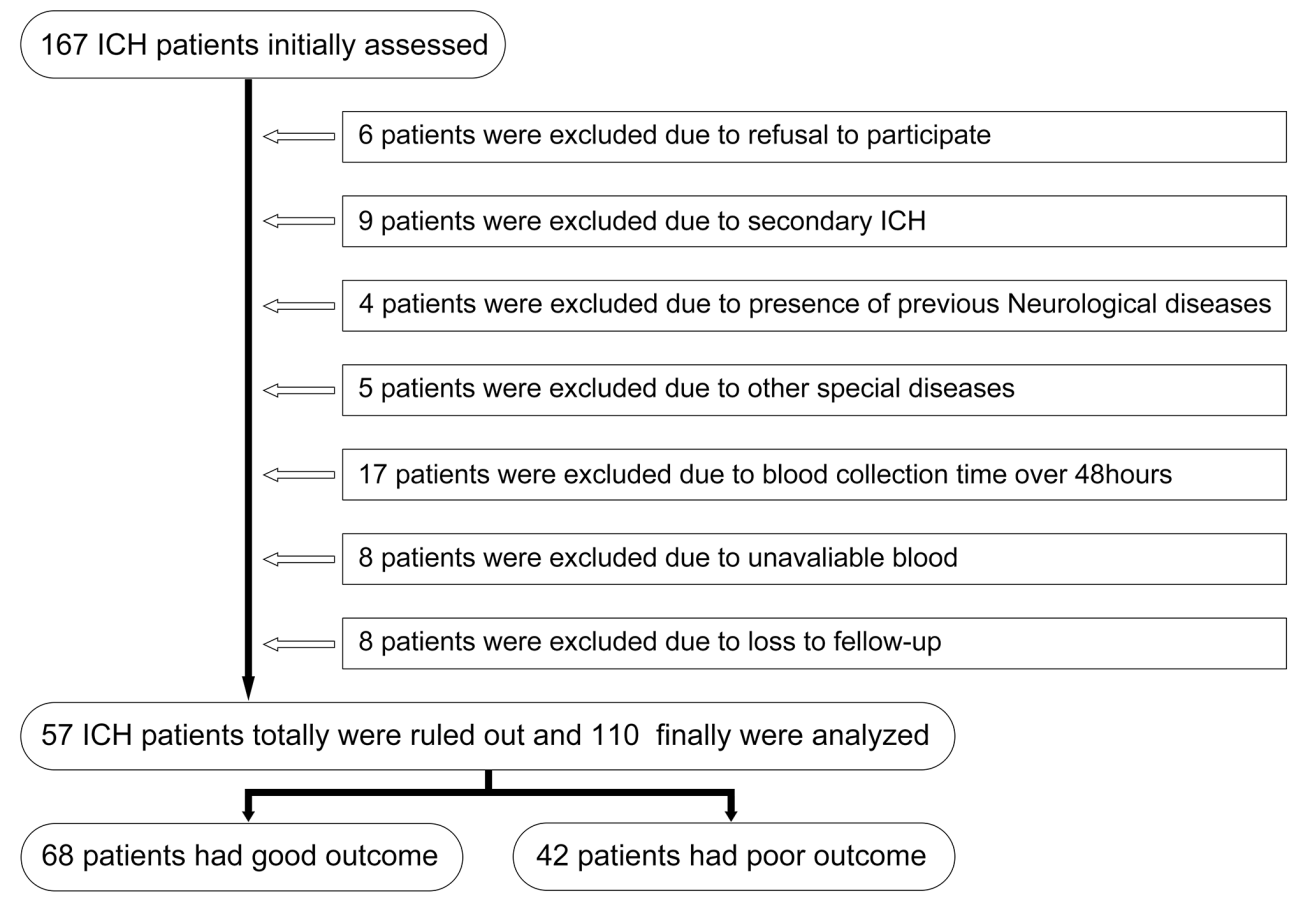
Flow chart for selecting eligible patients with acute spontaneous intracerebral hemorrhage. Initially, a total of 167 patients were assessed, 57 were then ruled out, and 110 were finally included for further analysis. Among these, 68 patients had good outcome and 42 patients had poor outcome Abbreviation: ICH, intracerebral hemorrhage

A total of 167 patients, who were admitted for conservative treatments after the onset of clinical symptoms, were initially assessed, then 57 cases were excluded following exclusion criteria, and 110 patients were finally analyzed in this study (Fig. 1). In the ICH group, there were 63 males and 47 females (male-to-female ratio, 1.34:1), who were aged from 25 to 92 years (mean, 66.9 years; standard deviation, 14.8 years). Also, there were 34 cases (30.9%) of current smoking and 27 cases (24.5%) of alcohol consumption. In addition, 110 healthy subjects constituted the control group. In controls, there were 59 males and 51 females (male-to-female ratio, 1.16:1), who were aged from 24 to 91 years (mean, 64.5 years; standard deviation, 16.2 years). In addition, there were 31 cases (28.2%) of current smoking and 24 cases (21.8%) of alcohol consumption. There were no statistically significant differences between the ICH group and the healthy control group in terms of age (*P* = 0.257), gender percentage (*P* = 0.587), current smoking (*P* = 0.658) and alcohol consumption (*P* = 0.632).

In the ICH group, history of illness included 78 cases (70.9%) of hypertension and 24 cases (21.8%) of diabetes mellitus. Moreover, time from stroke to admission ranged from 2.2 to 23.6 h (median, 9.1 h, upper-lower quartiles; 6.1 to 14.5 h), time between stroke and blood collection varied from 2.5 to 24.0 h (median, 9.4 h; upper-lower quartiles, 6.4 to 15.0 h), and systolic arterial pressure and diastolic arterial pressure changed from 104 to 199 mmHg (mean, 145.4 mmHg; standard deviation, 20.1 mmHg) and from 51 to 122 mmHg (mean, 83.0 mmHg; standard deviation, 13.8 mmHg) respectively. Laboratory tests showed that blood glucose levels ranged from 3.4 to 24.2 mmol/L (median, 6.8 mmol/L; upper-lower quartiles, 5.6 to 8.7 mmol/L), and the range of blood leucocyte count was from 2.7 to 22.4 × 10^9^/L (median, 7.7 × 10^9^/L; upper-lower quartiles, 5.7 to 10.8 × 10^9^/L). Some blood electrolytes, such as sodium and potassium, were also tested, and their levels ranged from 121.0 to 148.0 mmol/L (median, 141.0 mmol/L; upper-lower quartiles, 139.0 to 142.0 mmol/L) and from 2.7 to 5.2 mmol/L (median, 3.7 mmol/L; upper-lower quartiles, 3.4 to 4.0 mmol/L).

GCS scores were used to evaluate the severity of acute brain injury after ICH, which ranged from 3 to 15 (median, 13; upper-lower quartiles, 11 to 14). Based on GCS, the scores 13–15, 9–12 and 3–8 were found in 66 (60.0%), 34 (30.9%) and 10 (9.1%) patients respectively. With respect to some radiological parameters, there were 15 cases (13.6%) of infratentorial hemorrhage and 22 cases (20.0%) of intraventricular hemorrhage, and hematoma volumes ranged from 0.1 to 76.6 mL (median, 13.8 mL; upper-lower quartiles, 5.5 to 23.5 mL). GOS was a scoring scheme to assess 90-day prognosis after ICH (median, 4; upper-lower quartiles, 3 to 4), the scores of 1, 2, 3, 4 and 5 were revealed in 9, 7, 26, 45 and 23 cases respectively. In other words, scores of 1 to 3 were defined as poor outcome (42 cases, 38.2%) and good outcome consisted of 4 and 5 (68 cases, 62.8%).

### Correlation of serum secretoneurin levels with stroke severity

**Fig. 2:**
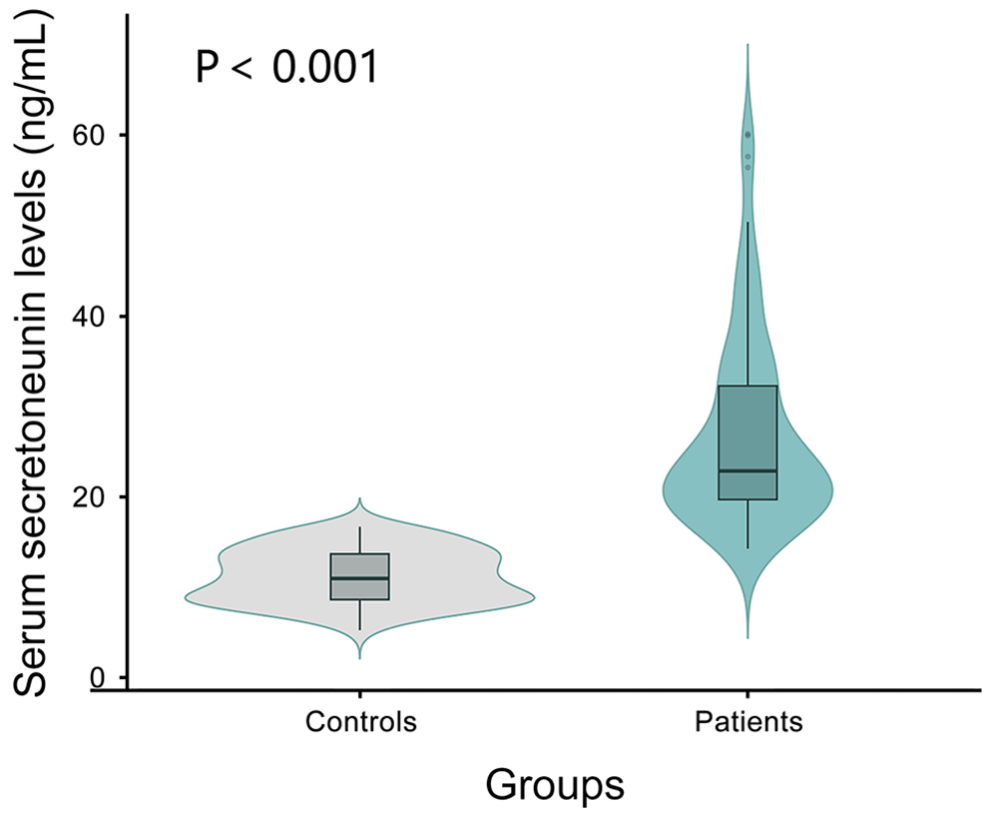
Levels of serum secretoneurin in different groups. There were significant differences in terms of serum secretoneurin levels between healthy controls and patients with intracerebral hemorrhage. Serum secretoneurin levels were significantly higher in patients than in healthy controls using the Mann–Whitney U test (*P* < 0.001). Serum secretoneurin levels were reported as median (upper-lower quartiles) in the figure

Serum Secretoneurin levels in ICH and healthy control groups ranged from 14.4 to 60.1 ng/mL (median, 23.0 ng/mL; upper-lower quartiles, 19.8 to 32.4 ng/mL) and from 5.5 to 10.1 ng/mL (median, 10.1 ng/mL; upper-lower quartiles, 9.3 to 10.9 ng/mL) respectively. Using Mann–Whitney U-test, serum secretoneurin levels of patients were obviously higher than those of controls (*P* < 0.001; Fig. 2).

**Fig. 3:**
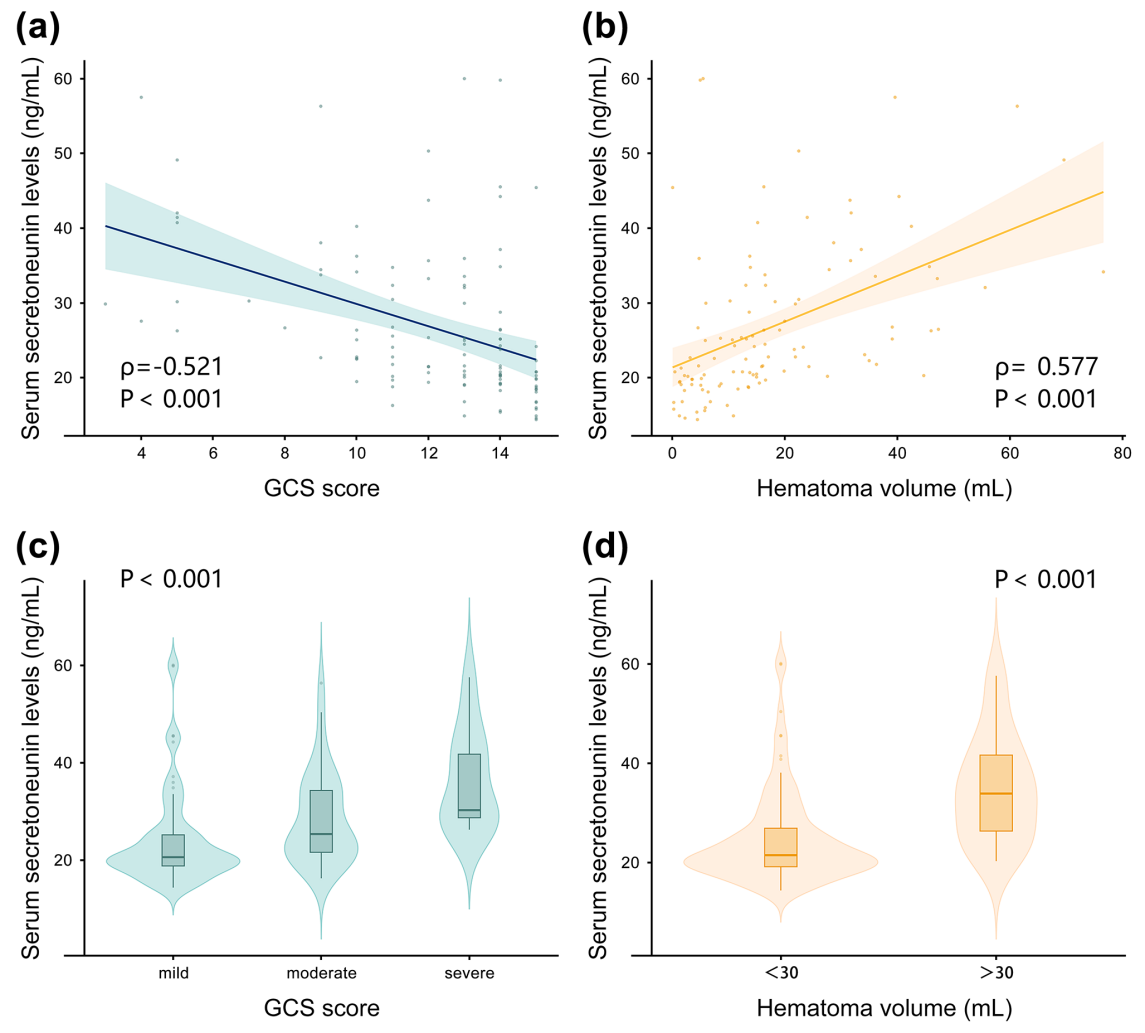
Relationship between serum secretoneurin levels and severity of hemorrhage after acute intracerebral hemorrhage. (**a**): Correlative analysis of serum secretoneurin levels with Glasgow Coma Scale score after acute intracerebral hemorrhage. Serum secretoneurin levels were strongly correlated with Glasgow Coma Scale score following stroke using the Spearman’s correlation coefficient (*P* < 0.001). (**b**): Relation of serum secretoneurin levels to hematoma volume after acute intracerebral hemorrhage. Serum secretoneurin levels were significantly enhanced with increased hematoma volume using Spearman correlation coefficients (*P* < 0.001). (**c**): Comparisons of serum secretoneurin levels across severity grade among patients with acute intracerebral hemorrhage. Significant differences in serum secretoneurin levels existed after stroke among multiple groups using the Kruskal − Wallis test (*P* < 0.001). (**d**): Differences of serum secretoneurin levels by hematoma volume after acute intracerebral hemorrhage. Patients with hematoma volume above 30 mL had substantially higher serum secretoneurin levels than those with hematoma volume below 30 mL using Mann–Whitney U-test (*P* < 0.001) Abbreviation: GCS, Glasgow Coma Scale

For the sake of justifying the relation between serum secretoneurin levels and hemorrhage severity, both GCS and hematoma volume were identified as the continuous variables, and also as the categorical variables. In Fig. 3a, there was a significantly negative correlation between serum secretoneurin levels and GCS scores (*P* < 0.001). Also, serum secretoneurin levels were substantially positively related to hematoma volume (*P* < 0.001; Fig. 3b). Alternatively, the levels were markedly highest in patients with GCS scores 3–8, followed by the scores 9–12, and were significantly lowest in those with the scores 13–15 (*P* < 0.001; Fig. 3c). Moreover, the levels were dramatically higher in patients with hematoma volume > 30mL than in the other remainders (*P* < 0.001; Fig. 3d).

To discover the potential relationships between serum secretoneurin levels and other variables, multiple factors were investigated. Intraventricular hemorrhage, GCS scores, hematoma volume and blood glucose levels were strongly correlated with serum secretoneurin levels (all *P* < 0.05; Table 1). The preceding four significant variables were forced into the multivariate linear regression model and subsequently, it was revealed that serum secretoneurin levels had independent correlations with GCS scores (beta, -0.267; t = -3.055; 95% CI, -1.553 to -0.330; VIF, 1.183; *P* = 0.003) and hematoma volume (beta, 0.315; t = 3.370; 95% CI, 0.085 to 0.330; VIF, 1.353; *P* = 0.001), but not with intraventricular hemorrhage (beta, 0.108; t = 1.219; 95% CI, -1.745 to -7.312; VIF, 1.219; *P* = 0.226) and blood glucose levels (beta, 0.136; t = 1.659; 95% CI, -0.082 to 0.918; VIF, 1.041; *P* = 0.100).

**Table 1:** Bivariate correlation analysis between serum secretoneurin levels and other variables in 110 intracerebral hemorrhage patients

Components	ρ	*P* value
Age (years) Gender (male/female) Hypertension Diabetes mellitus Current smoking Alcohol consumption Admission time (hours) Blood-collection time (hours) Systolic arterial pressure (mmHg) Diastolic arterial pressure (mmHg) infratentorial hemorrhage Intraventricular hemorrhage Glasgow Coma Scale score Hematoma volume (mL) Blood leucocyte count (×10^9^/L) Blood C-reactive protein (mg/L) Blood glucose levels (mmol/L) Blood sodium levels (mmol/L) Blood potassium levels (mmol/L)	0.055 0.027 -0.139 0.134 -0.143 -0.142 0.037 0.037 -0.100 -0.062 -0.098 0.302 -0.521 0.577 0.168 0.113 0.309 -0.151 -0.124	0.565 0.778 0.148 0.162 0.137 0.139 0.703 0.699 0.296 0.518 0.308 *0.001 *<0.001 *<0.001 0.079 0.240 *0.001 0.116 0.195

Notes: Correlations were done using Spearman Correlation Coefficient in intracerebral Hemorrhage. The asterisk indicates statistical significance (*P < 0.05)

### Relationship between serum secretoneurin levels and prognosis

**Fig. 4:**
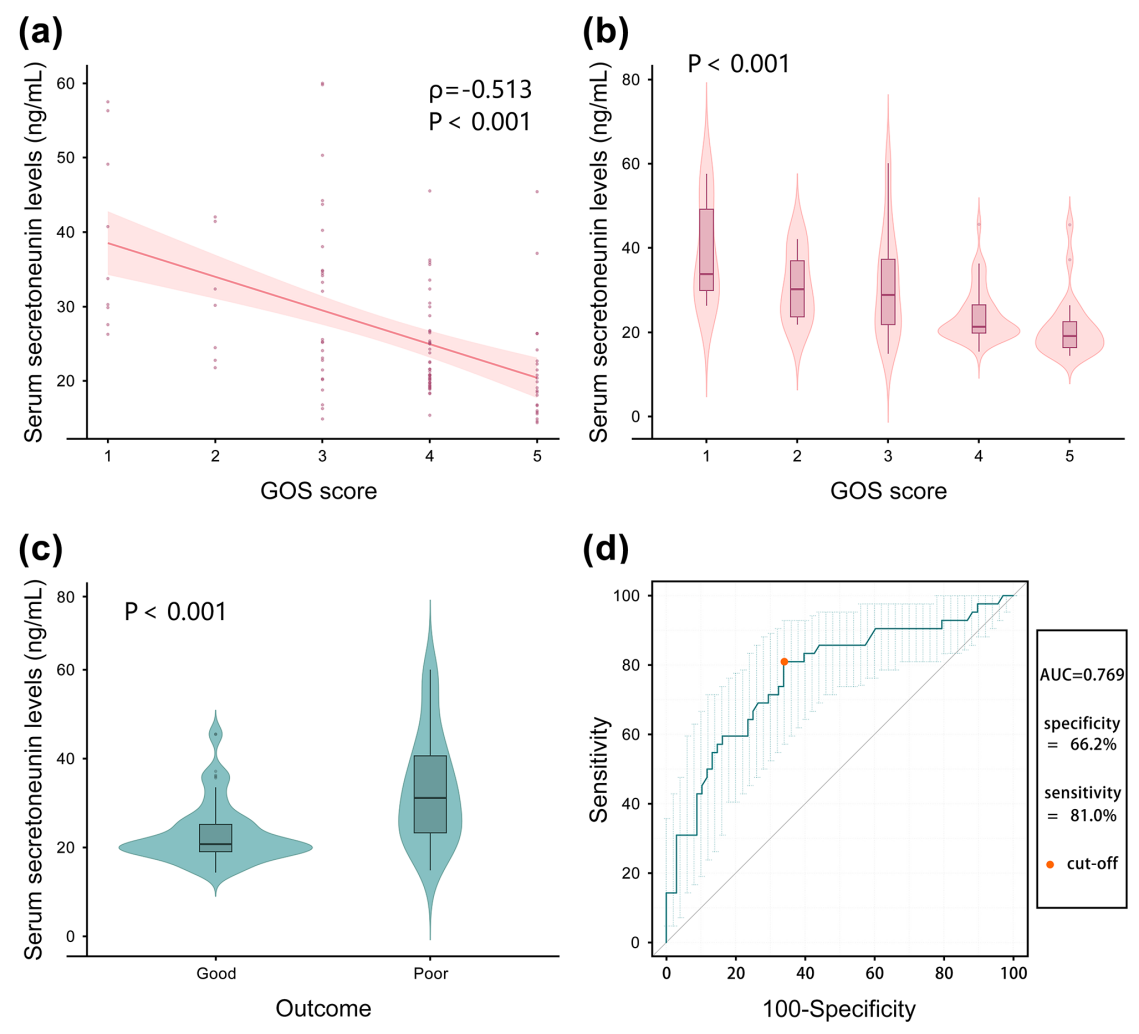
Relationship between serum secretoneurin levels and Glasgow Outcome Scale score after acute intracerebral hemorrhage. (**a**): Correlative analysis of serum secretoneurin levels with Glasgow Outcome Scale score after acute intracerebral hemorrhage. Serum secretoneurin levels were substantially negatively correlated with Glasgow Outcome Scale score following stroke using the Spearman’s correlation coefficient (*P* < 0.001). (**b**): Comparison of serum secretoneurin levels across outcome grade among patients with acute intracerebral hemorrhage. Significant differences in serum secretoneurin levels existed after stroke among subgroups using the Kruskal − Wallis test (*P* < 0.001). (**c**): Comparison of serum secretoneurin levels between patients with good outcome and those with poor outcome after intracerebral hemorrhage. Significant differences in serum secretoneurin levels existed after stroke in these two groups by the Mann–Whitney U test (*P* < 0.001). (**d**): Receiver operating characteristic curve analysis of serum secretoneurin levels. Serum secretoneurin levels conspicuously predicted a 90-day poor outcome after intracerebral hemorrhage (area under curve, 0.769; 95% confidence interval, 0.675–0.863). The Orange point represents the cut-off value of serum secretoneurin levels, and the value at this point is 22.8 ng/mL. According to this value, patients with poor prognosis after 90 days can be distinguished with specificity and sensitivity values of 66.2% and 81.0% (maximum Youden index J, 0.472) respectively. Two bars represent 95% confidence interval of area under curve Abbreviations: GOS, Glasgow Outcome Scale; AUC, area under curve

In Fig. 4a, serum secretoneurin levels were significantly correlated with GOS scores (*P* < 0.001). Likewise in Fig. 4b, serum secretoneurin levels were strongly decreased with the increasing GOS scores (*P* < 0.001). Also, the levels were substantially higher in patients with a poor prognosis than in those with a good prognosis (*P* < 0.001; Fig. 4c). Moreover, serum secretoneurin levels efficiently predicted a poor prognosis (AUC, 0.769; 95%CI, 0.675–0.863). Additionally, serum secretoneurin levels ≥ 22.8 ng/mL distinguished patients at risk of 90-day adverse prognosis with 66.2% sensitivity and 81.0% specificity (Youden index J = 0.472; Fig. 4d).

**Fig. 5:**
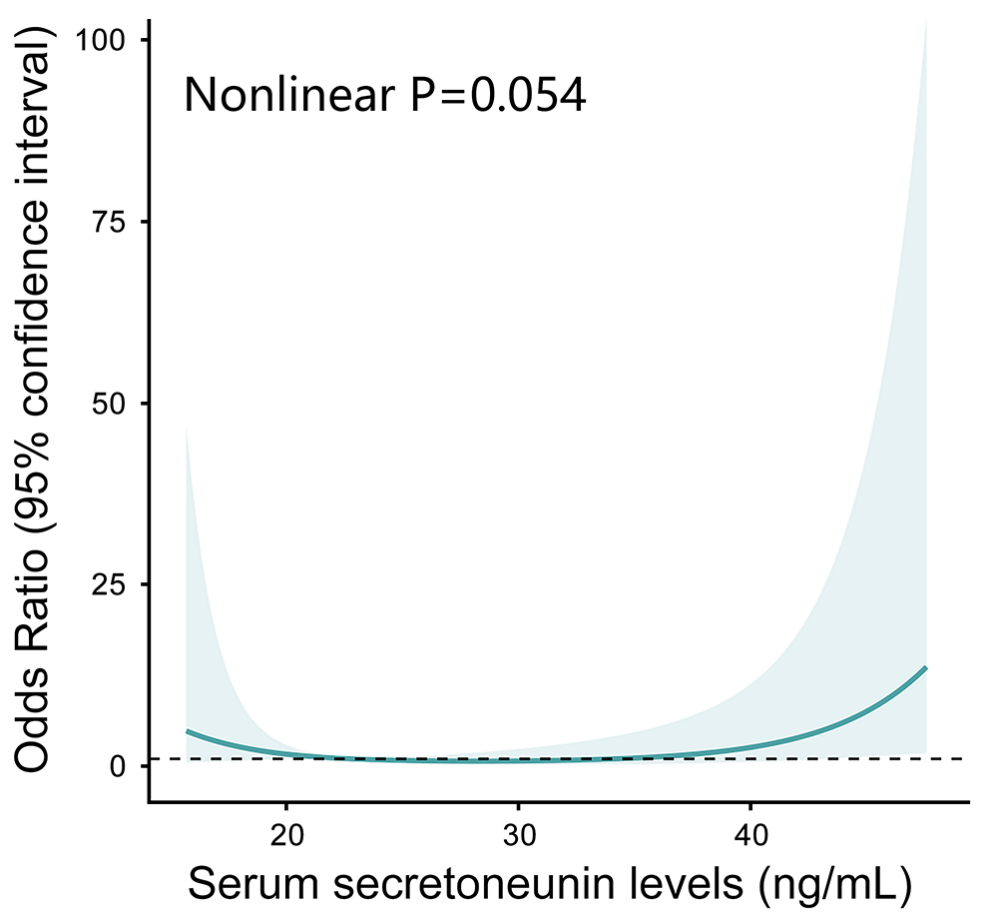
Restricted cubic spline models for the relationship between serum secretoneurin levels and risk of poor prognosis. *P* for nonlinear was 0.054. The 95% confidence interval of odds ratio was represented by the blue-shaded area

**Fig. 6:**
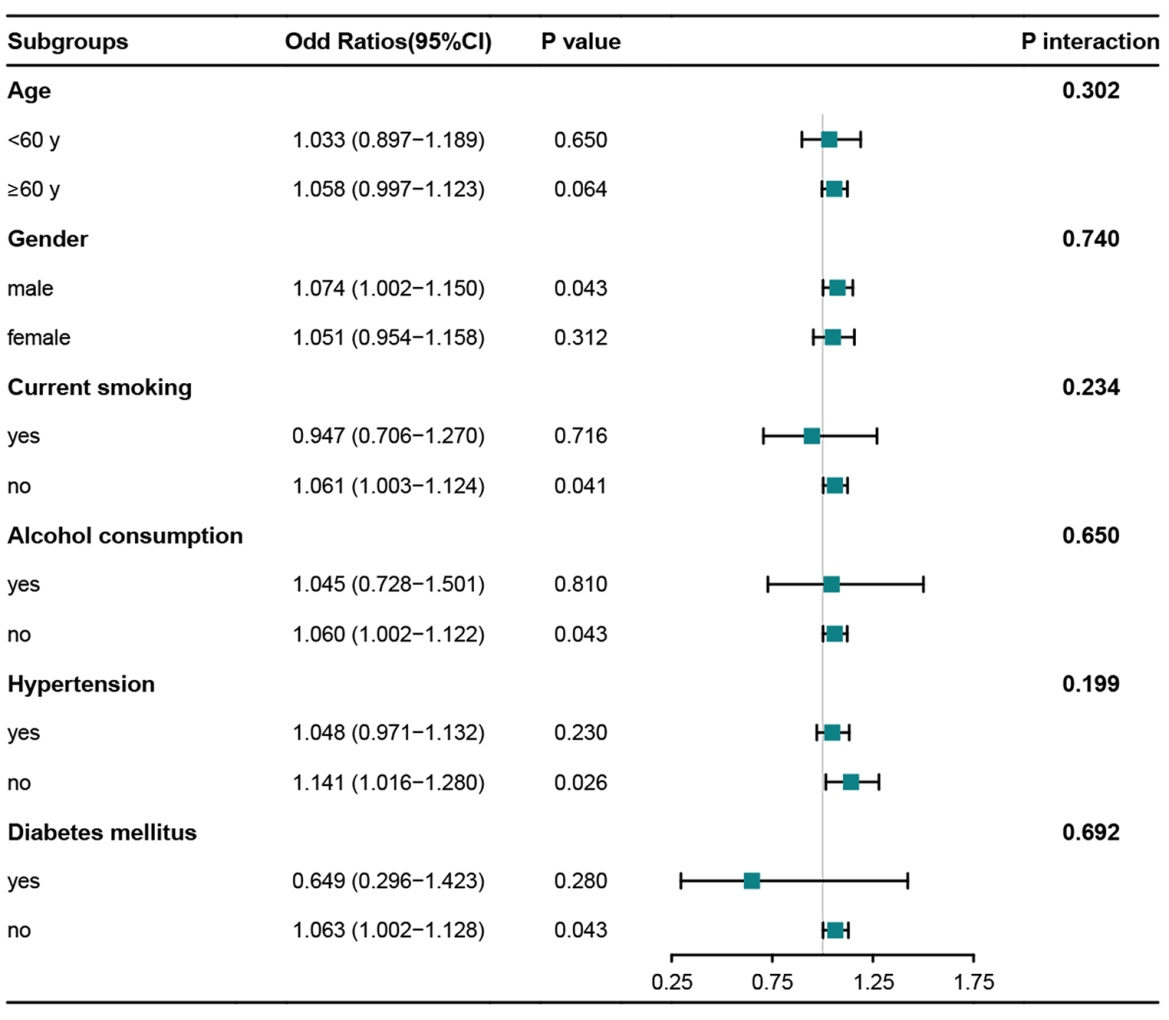
Forest plot subgroups associated with 90-day prognosis in patients with intracerebral hemorrhage. Serum secretoneurin levels in subgroups male, those never currently smoking, those negative for alcohol consumption, those had no hypertension, and those without diabetes mellitus had predictive benefits for poor prognosis(all *P* < 0.05). There was no statistical difference between subgroups (P interaction > 0.05) Abbreviation: CI, confidence interval

**Fig. 7:**
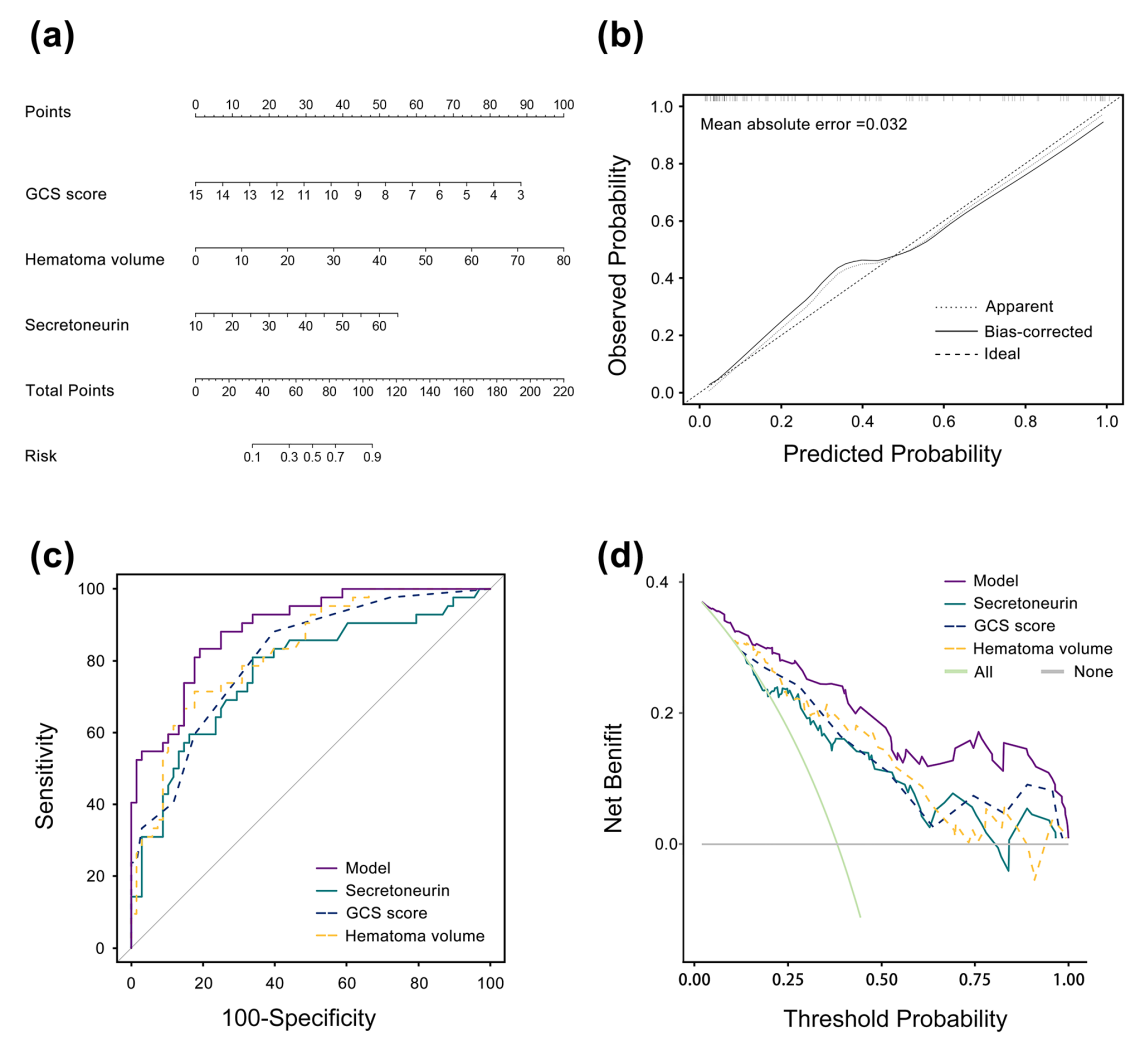
The prediction model for poor outcome in intracerebral hemorrhage. (**a**): The prognosis of patients with intracerebral hemorrhage after 90 days can be accurately predicted by the nomogram. (**b**): Calibration curves of 90-day prognosis for intracerebral hemorrhage patients. Mean absolute error was 0.032, and represented the reliability of the nomogram. (**c**): Comparison of the abilities among serum secretoneurin levels, Glasgow Coma Scale, hematoma volume and prediction model for forecasting the poor outcome with intracerebral hemorrhage after 90 days. Prognostic predictive ability of serum secretoneurin levels (area under curve, 0.769; 95% confidence interval, 0.674–0.864) was similar to those of Glasgow Coma Scale score (area under curve, 0.809; 95% confidence interval, 0.729–0.889; *P* = 0.468) and hematoma volume (area under curve, 0.825; 95% confidence interval, 0.748–0.903; *P* = 0.259), but prediction model had a huge boost with them(area under curve, 0.891; 95% confidence interval, 0.832–0.950; both *P* < 0.05). (**d**): Decision curve analysis of 90-day prognosis for intracerebral hemorrhage patients Abbreviation: GCS, Glasgow Coma Scale

To clarify the relationship between serum secretoneurin levels and the risk of adverse prognosis, RCS analysis was carried out. In Fig. 5, we found that there was a linear association between serum secretoneurin levels and the risk of poor outcome (*P* > 0.05). Table 2 shows that hematoma volume, blood glucose and serum secretoneurin levels of patients with poor outcome were significantly higher than those of patients with good outcome (all *P* < 0.05). While GCS scores were significantly lower in patients with development of poor outcome than in those with good outcome (*P* < 0.05). In addition, patients with poor outcome tended to have a significantly higher percentage of intraventricular hemorrhage (*P* < 0.05). Those significant factors on univariate analysis were incorporated in multivariate logistic regression analysis, and then we found that serum secretoneurin levels (odd ratio, 1.061; 95%CI, 1.004–1.122; *P* = 0.037), GCS (odd ratio, 0.639; 95%CI, 0.490–0.832; *P* < 0.001) and hematoma volume (odd ratio, 1.079; 95%CI, 1.033–1.127; *P* < 0.001) were the independent predictors of poor outcome. There were no significant differences in its association with intraventricular hemorrhage (odd ratio, 1.089; 95% CI, 0.266–4.452; *P* = 0.906) and blood glucose levels (odd ratio, 1.031; 95%CI, 0.878–1.211; *P* = 0.706). In addition, using subgroup analysis, there were no significant interactions between serum secretoneurin levels and other variables, such as age, gender, hypertension, diabetes mellitus, current smoking and alcohol consumption (all *P* interaction > 0.05; Fig. 6). A nomogram was constructed based on the independent predictive factors, including serum secretoneurin levels, GCS and hematoma volume. Figure 7a shows that the individual scores and their total scores could visualize the risk of ICH at 90 days in the nomogram. As expected, the AUC of the prediction model was up to 0.891 (95%CI, 0.832–0.950; Fig. 7b). The model’s discriminative ability was significantly higher, as compared with GCS scores (AUC, 0.809; 95% CI, 0.729–0.889; *P* = 0.028) and hematoma volume (AUC, 0.825; 95% CI, 0.748–0.903; *P* = 0.022). Under calibration curve (Fig. 7c), a reliable result was obtained (mean absolute error < 0.05). Moreover, decision curve analysis was used to compare clinical benefits between single factors and the prediction model (Fig. 7d). The results showed that the prediction model added more net benefit in all patients compared to those independent risk factors.

**Table 2:** Demographic, clinical, radiological and biochemical factors for 90-day poor outcome after acute intracerebral hemorrhage

Components	Poor outcome	Good outcome	*P* value
Number	42 (38.2%)	68 (61.8%)	
Age (years) Gender (male/female) Hypertension Diabetes mellitus Current smoking Alcohol consumption Admission time (hours) Blood-collection time (hours) Systolic arterial pressure (mmHg) Diastolic arterial pressure (mmHg) Infratentorial hemorrhage Intraventricular hemorrhage Glasgow Coma Scale score Hematoma volume (mL) Blood leucocyte count (×10^9^/L) Blood C-reactive protein (mg/L) Blood glucose levels (mmol/L) Blood sodium levels (mmol/L) Blood potassium levels (mmol/L) Serum secretoneurin levels (ng/mL)	69.9 ± 16.2 23/19 30 (71.4%) 10 (23.8%) 10 (23.8%) 8 (19.0%) 7.9 (5.8–12.2) 8.3 (6.1–12.5) 145.4 ± 21.8 79.9 ± 12.9 8 (19.0%) 13 (30.9%) 11 (9–13) 23.7 (14.2–39.8) 8.1 (5.9–11.0) 2.5 (0.8-6.0) 7.5 (6.2–9.5) 140 (136–143) 3.6 (3.3-4.0) 31.2 (23.0–41.0)	65.0 ± 13.6 40/28 48 (70.6%) 14 (20.6%) 24 (35.3%) 19 (27.9%) 10.3 (6.3–17.7) 10.8 (6.8–18.2) 145.5 ± 19.1 84.9 ± 14.1 7 (10.3%) 9 (13.2%) 14 (12–15) 8.6 (3.5–15.4) 7.4 (5.7–10.5) 1.4 (0.5–3.6) 6.5 (5.5–8.1) 141 (139–142) 3.7 (3.4-4.0) 20.8 (19.1–25.4)	0.092 0.676 0.925 0.691 0.205 0.292 0.125 0.131 0.990 0.063 0.194 *0.024 *<0.001 *<0.001 0.597 0.066 *0.028 0.089 0.433 *<0.001

Notes: Quantitative data were reported as medians with 25th-75th percentiles or the mean ± standard deviation as appropriate. Qualitative data were presented as counts (proportions). Intergroup comparisons of various variables were performed using the χ2 test or Fisher’s exact test for qualitative data, and T test or Mann-Whitney U-test for quantitative data. Glasgow Outcome Scale score of 1–3 was designated as poor outcome. The asterisk indicates statistical significance (*P < 0.05)

## Discussion

Stroke, an acute disease of the central nervous system, is characterized by high morbidity and high mortality. Severity assessment and prognosis prediction are of great importance for treating patients with stroke [16]. Laboratory indexes can aid in disease diagnosis and outcome prediction. For decades, biomarkers have been paid extensive attention in ICH-related studies [17].

Secretoneurin expressions are substantially up-regulated under hypoxic conditions [18]. Functionally, secretoneurin acts as a protective factor characterized by angiogenesis and postnatal vasculogenesis [7]. Its molecular mechanisms are rather complex. In a study using secretoneurin, skeletal muscle cells were regenerated after hypoxia by a nitric oxide-dependent mechanism [19]. In another study about experimental ischemic stroke, secretoneurin protected neurons against hypoxic injury obviously by activating vascular regeneration Jak2/Stat3 pathway [8]. In mice with cerebral ischemia, secretoneurin expressions in hippocampus region were significantly elevated, with the peak on the second day after ischemia. Also, blood secretoneurin levels were significantly elevated in rats after excitotoxic brain injury [20]. Interestingly, levels of blood secretoneurin were markedly increased in several human neurological disorders, such as ischemic stroke, Alzheimer’s disease and neonatal hypoxic-ischemic brain injury [9, 11, 12]. Consistently, in our study, serum secretoneurin levels were markedly enhanced after ICH. Overall, it is undoubted that blood secretoneurin levels may be greatly raised after acute or chronic brain injury.

Accumulating evidence has shown that secretoneurin may be a novel prognostic biomarker in cardiovascular diseases [21, 22]. In recent years, secretoneurin has been gradually recognized as a potential biomarker in some neurological diseases [23]. Specifically, umbilical cord secretoneurin levels were highly correlated with neurodevelopmental outcome of hypoxia-ischemia brain injury of neonates [24]. Alternatively, in patients with hypoxic brain injury after cardiopulmonary resuscitation, serum secretoneurin levels may be a promising early biomarker for predicting neurological outcome after hypoxic brain injury [25]. Also, there were high levels of serum secretoneurin in patients with traumatic brain injury; and serum secretoneurin levels were negatively related to GCS and GOS [13]. Moreover, another clinical study showed that serum secretoneurin was a reliable diagnostic biomarker for ischemic stroke patients, but was unsuccessful in manifesting the relationship between neurological outcomes and prognosis [12]. Thus, circulating secretoneurin may be a prognostic biomarker of acute brain injury.

Given that the preceding results of ischemic stroke were generated using univariate analysis, several multivariate analyses were performed in our study. We found that serum secretoneurin levels were markedly higher in patients with poor outcome than in those with good outcome after ICH. Also, serum secretoneurin levels were highly correlated with GCS scores and hematoma volume. Furthermore, the results using multivariate analysis were strongly supportive of the notion that serum secretoneurin levels may be independently correlated with GCS scores and hematoma volume, as well as have an independent relation to poor outcome at 90 days after ICH. It is worth noting that serum secretoneurin levels had a similar prognostic function to GCS and hematoma volume. To further explore the predictive ability of serum secretoneurin levels, a prediction model was constructed based on the independent predictive factors, including serum secretoneurin levels, GCS and hematoma volume. Interestingly, we found no substantial interactions between serum secretoneurin levels and the other variables, such as age, gender and chronic diseases. Area under curve of model was significantly higher than those of the conventional severity indicators (GCS and hematoma volume). Stability and accuracy of the model were satisfactorily demonstrated by calibration curve and decision curve. The above data are highly indicative of postulation that serum secretoneurin levels may take possession of efficient discriminative ability and serum secretoneurin may be a meaningful biomarker for the prognosis of ICH.

There are several strengths and weaknesses in the current study. The strengths are that (1) to the best of our knowledge, this study may for the first time report that serum secretoneurin levels could be significantly elevated in patients with ICH and serum secretoneurin levels could efficiently predict the prognosis of ICH; (2) relationships between serum secretoneurin levels and severity in addition to poor prognosis were verified using multivariate analysis, meaning high reliability and scientificity in our conclusions. The weaknesses are as follows. First, because all patients in this study underwent conservative treatments, it is unclear about how to evacuation of hematoma affects serum secretoneurin levels after ICH. Compelling evidence has shown that secretoneurin levels were tremendously expressed under states of tissue hypoxia [18], and serum secretoneurin levels were significantly correlated with GCS and hematoma volume in the current study. Hence, it is inferred that serum secretoneurin levels may be reduced with hematoma evacuation in ICH. However, it may be of great importance that a group of patients in need of hematoma evacuation will be enrolled in the future to investigate the effect of hematoma evacuation on serum secretoneurin levels after ICH. Second, temporal changes in serum secretoneurin levels were not determined after ICH. Third, our conclusions were made based on a medium number of patients and therefore a large cohort study would be needed to validate the reliability of serum secretoneurin as a predictor of poor prognosis in ICH.

## Conclusion

To the best of our knowledge, it is for the first time verified that (1) significantly elevated serum secretoneurin levels after ICH, in independent correlation with GCS and hematoma volume, are independently predictive of 90-day post-stroke adverse outcome; (2) serum secretoneurin levels had the similar prognostic predictive ability to GCS and hematoma volume under ROC curve; and (3) prognosis prediction model, in which serum secretoneurin levels, GCS and hematoma volume were incorporated, performs efficiently under ROC curve, calibration curve and decision curve. In summary, serum secretoneurin may be a useful biomarker for assessing ICH severity and predicting the adverse prognosis of ICH.

## Data Availability

The data that support the findings of this study are available from the corresponding author, [Xin Zhang], on special request.

## Abbreviations

ICH: Intracerebral hemorrhage
GCS: Glasgow Coma Scale
CT: Computerized tomography
GOS: Glasgow Outcome Scale
ELISA: Enzyme-linked Immunosorbent Assay
OR: Odds ratio
95% CI: 95% confidence interval
ROC: Receiver operating characteristic
AUC: Area under curve
RCS: Restricted cubic spline
